# A Prospective Observational Study on the Tolerability of Transnasal Bronchoscopy With a Surgical Mask for Aerosol Control

**DOI:** 10.7759/cureus.28197

**Published:** 2022-08-20

**Authors:** Yusuke Usui, Shinji Sasada, Keisuke Kirita, Sayaka Nagayama, Saori Murata, Yumi Tsuchiya, Kota Ishioka, Saeko Takahashi, Morio Nakamura, Kazuma Kishi

**Affiliations:** 1 Pulmonary Medicine, Tokyo Saiseikai Central Hospital, Tokyo, JPN; 2 Division of Respiratory Medicine, Department of Internal Medicine, Toho University Graduate School of Medicine, Tokyo, JPN; 3 Department of Pulmonology, The Fraternity Memorial Hospital, Tokyo, JPN

**Keywords:** flexible bronchoscopy, cough frequency, pain score questionnaire, aerosol control, covid-19, trans-nasal bronchoscopy with surgical mask

## Abstract

Background

As far as we know, there are no reports comparing the safety and cough frequency of transnasal bronchoscopy (TNB) with transoral bronchoscopy (TOB).

Methods

The subjects were 50 patients who underwent either TNB or TOB and completed the pain score questionnaire between May and November 2020. Complications, pain scores, and cough frequency (times per minute) were compared between the patients with TNB and TOB. A surgical mask was worn over the mouthpiece during the examination.

Results

Thirty-two and 18 patients underwent TNB and TOB, respectively. Between the two groups, there were no significant differences in examination time and frequency of serious complications. In pain scores, there were no significant differences in terms of anesthesia suffering, several pains during the examination, and availability of re-examination. The TNB group did not feel the prolonged examination time compared to the TOB group (p=0.04). Cough frequency was lower in the TNB group than in the TOB group (0.36 vs 0.73, p=0.027). Moreover, cough frequency in the 25 TNB patients who underwent thin bronchoscopy was significantly lower (0.19 vs 0.73, p<0.01).

Conclusions

TNB with a surgical mask was well tolerated and safe. Cough frequency in the transnasal thin bronchoscopy was extremely low, suggesting aerosol reduction can be expected.

## Introduction

The new coronavirus infection (COVID-19) has been reported to have caused 490,000 infections and 9,200 deaths in Japan as of April 2021 [1]. Although the number of deaths is lower than in Europe and the United States, medical institutions have been burdened by the lack of personal protective equipment and nosocomial infections. In addition, when bronchoscopy is performed on COVID-19 positive patients or suspected patients, relevant academic societies have to be notified to consider the risk of infection to medical personnel due to aerosol generation. It is recommended that eye protection such as N95 masks or equivalent, long-sleeved gowns, gloves, goggles, or face shields be worn during treatment [2]. On the other hand, to minimize the aerosol effects of coughing during endoscopy, a method has been proposed in which the scope is inserted over a modified surgical mask worn by the patient [3] or a plastic cube barrier covering the patient's head [4]. By combining such devices, it is important not to postpone or avoid the diagnosis and treatment of respiratory diseases, especially lung cancer, which can be fatal if left untreated.

Bronchoscopy with a transnasal approach has been performed for a long time, but it has not been proven whether it causes less coughing, which is the cause of aerosol generation, compared to the transoral approach. In this study, we prospectively investigated the tolerability and patient distress of trans-nasal bronchoscopy with a surgical mask.

## Materials and methods

**Figure 1:**
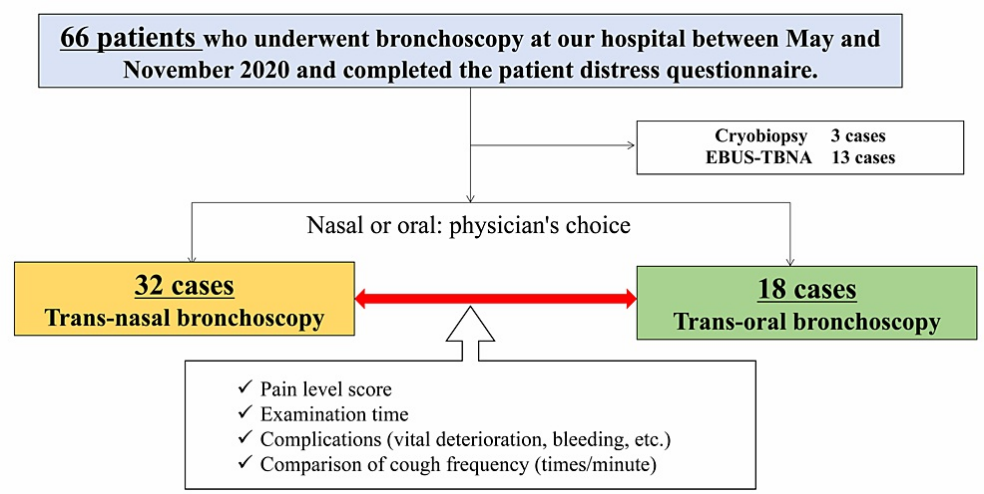
Study flow chart. Of the 66 patients who underwent bronchoscopy at our hospital between May 2020 and November 2020 and were eligible to complete the patient distress questionnaire, 50 were included, excluding those who underwent transbronchial lung cryobiopsy (TBLC) under an intubation tube, and those who underwent endobronchial ultrasound-guided transbronchial needle aspiration (EBUS-TBNA). The patients were divided into two groups, nasal (nasal group) and oral (oral group), and the examination parameters, patient distress scores, and frequency of coughing were compared between the two groups. The type of scope and the choice of nasal or oral approach were made at the discretion of the attending physician.

This study was a prospective observational study. A total of 66 patients who underwent bronchoscopy at our institution for the diagnosis of benign or malignant disease, differentiation of inflammation, or diffuse lung disease were included in this study between May 2020 and November 2020. They were eligible to complete the pain score questionnaire on bronchoscopy, excluding those who underwent transbronchial lung cryobiopsy (TBLC) under intubation and those who underwent endobronchial ultrasound-guided transbronchial needle aspiration (EBUS-TBNA) (Figure 1). All of these tests were performed on a standby basis, and emergency tests and bedside bronchoscopies were excluded. To assess patient parameters during the examination, the following data were recorded: baseline SpO_2_, minimum SpO_2_, change in SpO_2_, systolic blood pressure at the beginning of the examination, and minimum systolic blood pressure. The cough frequency and complications during the examination were also investigated. Complications in this study were defined as bleeding that forced interruption of the examination, pneumothorax (including those that did not require drainage) or pneumonia after the examination, and deterioration of vital signs to the point that ICU admission was necessary after the examination.

The patients were divided into two groups according to the insertion route of the scope: trans-nasal bronchoscopy group (TNB group) or trans-oral bronchoscopy group (TOB group). Examination parameters, patient pain scores, and cough frequency were compared between these two groups. The type of scope and the choice of nasal or oral approach were made at the discretion of the attending physician, but there were no clear criteria and no selection was made based on patient age, gender or body size, or underlying disease. However, patients who refused nasal endoscopy when obtaining prior consent, or who had difficulty with nasal approach due to nasal anatomy were approached orally.

To calculate the cough frequency (number of coughs/minute), examination time was calculated from the time when the scope is inserted and the time when it is removed. The cough count was done by an assistant in the endoscopy room. Since it was difficult to record the number of times in units of times, we used 0: 0 times, 1: 1 time, 2-3 times: 3 times, 4-5 times: 5 times, 6-10 times: 10 times, 11-29 times: 20 times, 30 or more times: 30 times. This study was approved by the Institutional Review Board of Tokyo Saiseikai Central Hospital (2020-010-1, approved on May 22, 2020). Written informed consent was obtained from each participant.

Preparation for examination

All doctors and nurses in a bronchoscopy room wore surgical masks over N95 masks, long-sleeved gowns, gloves, and goggles during the examination. From the viewpoint of a risk of aerosol generation, pharyngeal, laryngeal, and tracheal anesthesia using Jackson spray was not performed before the examination in all cases.

Methods of transnasal approach

**Figure 2:**
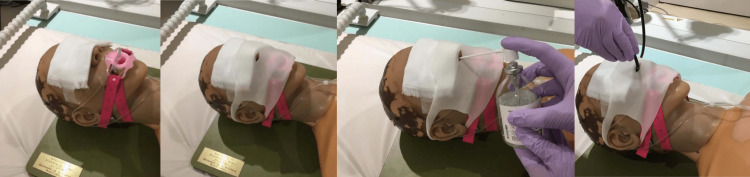
Nasal bronchoscopy procedure. In nasal bronchoscopy, an oxygen cannula is placed over a mouthpiece, and a surgical mask was used to cover the nose and mouth. For sedation, midazolam and pethidine were administered, followed by two sprays of naphazoline and lidocaine in each nasal cavity before the scope was inserted.

After administering midazolam and pethidine intravenously to a patient, the physician sprayed the left and right nostrils with naphazoline nitrate (approximately 0.5 ml in each nostril) and 8% lidocaine (2-3 times in each nostril). To prevent the spread of aerosols due to coughing during the examination, a surgical mask with holes was worn to cover the patient's mouth, and a scope was inserted after covering both eyes with gauze (figure2).

Methods of transoral approach

After intravenous administration of sedative and analgesic drugs as in the nasal route, 8% xylocaine was sprayed into the pharynx two to three times, a mouthpiece was attached, a cannula was placed in the nose, and oxygen was administered. Then, a surgical mask with a hole was attached, and the scope was inserted through the mouth.

Bronchoscope and pain score questionnaire used in this study

Three types of scopes, namely the BF-1TQ290 (scope tip OD: 5.9 mm), and BF-P290 (scope tip OD: 4.0mm) (Olympus Medical Systems, Tokyo, Japan), were used for this study. The pain score questionnaire used in this study was based on a nationwide questionnaire conducted by the Japanese Society of Respiratory Endoscopy in the past [5] where 1 was the best result and 5 was the worst result. The questionnaires were obtained at least 2 hours after bronchoscopy when the patient was fully awake.

Statistical analysis

Student's t-test was used to test continuous variables. Also, the variables were normally distributed. Categorical variables were compared using Fisher's exact test. P values <0.05 were considered statistically significant. All statistical analyses were performed using EZR, which is a statistical software that extends the functionality of R and R Commander and is available free of charge on the website of Saitama Medical Center, Jichi Medical University [6].

## Results

Of the 50 patients who completed the pain score questionnaire, the median age was 73 years (26-88 years), 32 (64.0%) were male and 18 (36.0%) were female. Of these, nasal bronchoscopy was performed in 32 cases (64.0%) and oral bronchoscopy in 18 cases (36.0%). There was no clear difference between the two groups in terms of age, gender, or test duration (Table 1). The total dose of anesthetics administered before and during the examination was higher in the TOB group than in the TNB group. In addition, the TNB group tended to have a lower oxygen flow rate and less fluctuation in SpO_2_ value during the examination than the TOB group (Table 1).

**Table 1:** Patient characteristics. There was no significant difference in age, gender, and examination time between in two groups. Doses of midazolam were significantly less in the TNB group. The TOB group tended to have lower oxygen flow rates and less fluctuation in SpO2 value during the examination than the TOB group. SD: standard deviation; SpO2: saturation of peripheral oxygen; TNB: transnasal bronchoscopy; TOB: transoral bronchoscopy

	TNB group ( n=32 )	TOB group ( n=18 )	p-value
Age (years) (median, range)	73 (32-88)	73 (26-87)	0.29
Sex, number of Males (n, %)	20 (71)	12 (43)	
Examination time (min) (median, range)	22 (3-40)	21 (7-43)	0.54
Dosage of anesthetics (mean±2SD )			
Midazolam (mg)	2.8 ± 0.70	3.6 ± 1.3	<0.001
Pethidine hydrochloride (mg)	17 ± 10	21 ± 7.5	0.24
Parameters during the examination (mean±2SD )			
Baseline SpO_2_ (%)	98 ± 1.6	98 ± 1.3	0.52
Minimum SpO_2_ (%)	96 ± 4.9	96 ± 3.8	0.58
SpO_2_ change (%)	-0.56 ± 5.1	-2.2 ± 3.9	0.24
Oxygen flow (L/min)	3.1 ± 0.73	3.5 ± 1.5	0.20
Baseline systolic blood pressure (mmHg)	145 ± 20	136 ± 28	0.21
Minimum systolic blood pressure during the examination (mmHg)	132 ± 22	123 ± 27	0.24

**Figure 3:**
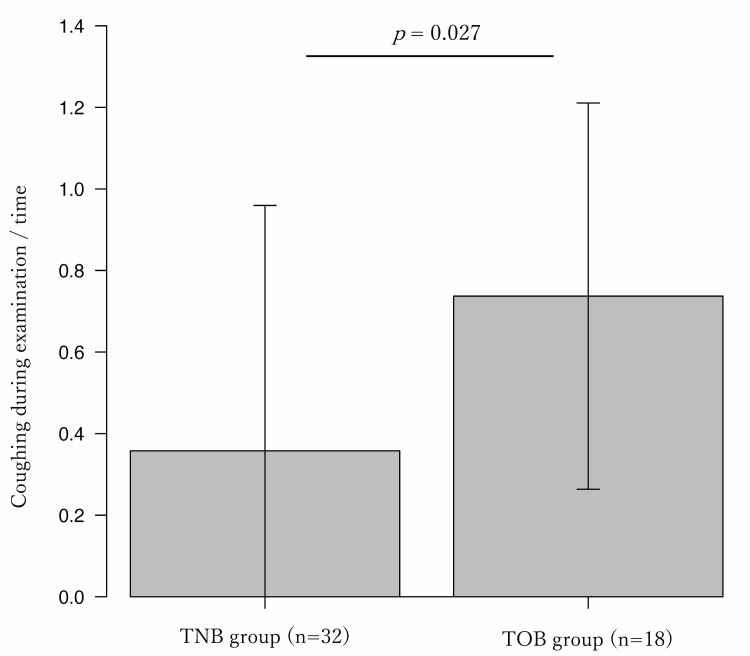
Comparison of cough frequency during examination. Cough frequency of TNB group was lower than TOB group (0.36 vs 0.73, p=0.027). TNB: transnasal bronchoscopy; TOB: transoral bronchoscopy

**Figure 4:**
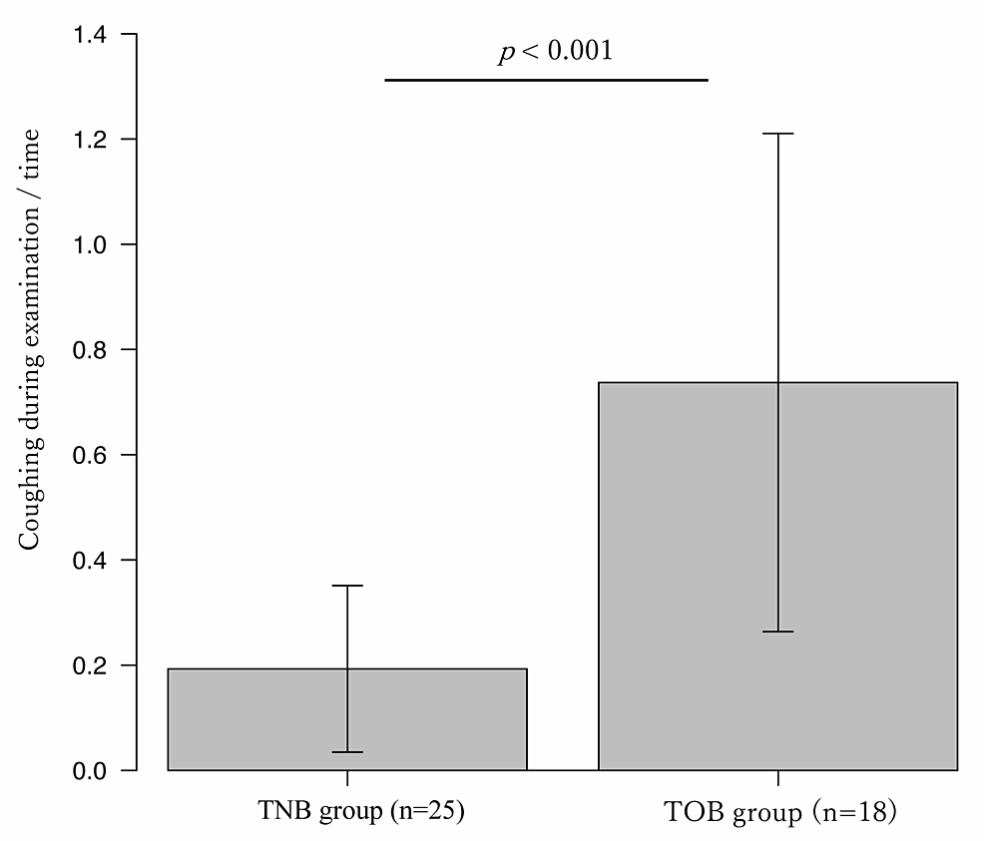
Comparison of cough frequency during examination. Among the TNB group, 25 patients who underwent thin diameter bronchoscope (Olympus BF-P290) was significantly lower than the TOB group (0.19 vs 0.73, p<0.001). TNB: transnasal bronchoscopy; TOB: transoral bronchoscopy

Complications included two cases of epistaxis in the nasal group, one case of vomiting, one case of tracheal hemorrhage that interrupted the examination, and one case of an asthma attack in the oral group. However, there was no significant difference in the frequency of complications between the two groups, and there were no serious complications requiring ICU admission or prolonged hospitalization after the examination. Nasal bleeding was observed in two patients in the TNB group, but the bleeding was stopped in both cases, and the examination could be continued by switching to transoral examination. As for the comparison of the scopes used in this study, the TNB group used a thin diameter scope and the TOB group used a procedural scope more frequently (Table 2). There was no significant difference in the content of the procedure between nasal and oral bronchoscopy, except that transbronchial lung biopsy (TBLB) using disposal mini cryoprobe (1.1 mm diameter) was performed more frequently in the TOB group (Table 3). A comparison of the pain score questionnaire showed that the TNB group felt the examination time was significantly shorter than the TOB group, and the remaining items were not significantly different between the two groups (Table 4). The cough frequency was significantly lower in the TNB group than in the TOB group (Figure 3). In addition, when the TNB group using a thin diameter scope was compared with the TOB group, the cough frequency was found to be even lower in the TNB group using a thin diameter scope (Figure 4).

**Table 2:** Bronchoscopes used for examination. Normal diameter bronchoscopes are frequently selected for a transoral approach, and thin bronchoscopes are used for a transnasal approach. Normal diameter bronchoscope: BF 1T260 and 1TQ290, Thin diameter bronchoscope: BF-P290; TNB: transnasal bronchoscopy; TOB: transoral bronchoscopy

	TNB group (n=32) n, %	TOB group (n=18) n, %	p-value
Use of normal diameter bronchoscope	7 (22%)	15 (83%)	＜ 0.001
Use of thin diameter bronchoscope	25 (78%)	3 (17%)	＜ 0.001

**Table 3:** Detail of the procedures. There was no significant difference between the two groups except for TBLB using disposal mini cryoprobe (1.1mm diameter). TNB: transnasal bronchoscopy; TOB: transoral bronchoscopy; EBUS-GS, endobronchial ultrasonography with a guide sheath; TBB, transbronchial biopsy: BAL, bronchoalveolar lavage; TBLB, transbronchial lung biopsy

	TNB group (n=32) n, %	TOB group (n=18) n, %	p value
TBB with EBUS-GS	21 (66)	13 (72)	0.76
TBB without EBUS-GS	1 (3.1)	2 (11)	0.13
BAL	2 (6.3)	4 (22)	0.17
Brushing	4 (13)	5 (26)	0.25
Washing	8 (25)	1 (56)	0.13
TBLB using mini cryoprobe	0 (0)	4 (22)	0.01
Others	1 (3.1)	1 (5.6)	

**Table 4:** Comparison of pain scores. There were no significant differences between the two groups in terms of anesthesia suffering, several pains during the examination, and the availability of re-examination. The TNB group did not feel the prolonged examination time compared to TOB group. TNB: transnasal bronchoscopy; TOB: transoral bronchoscopy

	TNB group (n=32)	TOB group (n=18)	p-value
Suffering from anesthesia	1.55 ± 0.96	1.33 ± 0.77	0.37
Memory during the examination	1.69 ± 1.28	1.94 ± 1.39	0.39
Pain during the examination	1.26 ± 0.68	1.11 ± 0.32	0.60
Suffering during examination	1.29 ± 0.64	1.61 ± 0.85	0.14
Length of examination time	1.13 ± 0.43	1.56 ± 0.86	0.035
Availability of re-examination	1.68 ± 1.25	1.33 ± 0.97	0.32

## Discussion

In this study, to minimize the spread of droplets during the examination, a patient wore a surgical mask to cover the mouth and nose, and the bronchoscope was inserted through a hole in the mask. With those conditions, we sought to determine the safety and tolerability of nasal bronchoscopy by comparing the pain score and frequency of complications of nasal bronchoscopy with oral bronchoscopy. As a result, the pain score level of the TNB group was comparable to that of the TOB group, and no serious complication was observed. The cough frequency of TNB group tended to be lower than that of the TOB group. In addition, the cough frequency of transnasal thin bronchoscopy was significantly lower than in that of the TOB group. Despite the lack of pharyngeal anesthesia prior to examination with the Jackson spray, the availability of retesting on the score was good. TNB with intravenous anesthesia using midazolam and pethidine seemed to be well tolerated.

Tolerability of transnasal bronchoscopy

Nasal bleeding is a typical complication of transnasal endoscopy, and previous studies reported that nasal bleeding occurred at a frequency of 2.7-7.0% [7-10]. In a retrospective study of upper gastrointestinal endoscopy in which patients were randomly assigned to TNB or TOB, nasal bleeding was reported to occur in 4% of patients [11]. Therefore, the frequency of nasal bleeding in TNB and gastrointestinal endoscopy was considered to be similar. In the present study, nasal bleeding occurred in 7.1% of patients. Although all bleeding could be stopped, the frequency of nasal bleeding in this study was higher than previously studied. This was probably because that our hospital was not accustomed to TNB, and therefore the scope damaged the nasal mucosa when trying to force it through the narrow nasal cavity. We should also pay attention to bleeding due to scope contact in the Kiesselbach area, where the anterior and posterior ethmoid and palatine artery branches form a dense vascular network. In addition, there is no consensus on how to choose between nasal and oral approaches to bronchoscopy, and there is no unified view on patient tolerability [12]. Some reports indicate that factors such as racial differences in nasal cavity volume, scope type, sedation procedure [4], severe anxiety before bronchoscopy, and bronchoscopist’s experience [13] affect the tolerability. From the results of the comparison of pain scores, the TNB group was considered to be as well tolerated as the TOB group. 

Tolerability of omission of pharyngeal anesthesia

In Japan, pharyngeal anesthesia with Jackson spray and intravenous sedation have been widely used as a pretreatment before bronchoscopy [14]. However, In a previous report examining the patient’s pain score questionnaire of intravenous anesthesia with midazolam and pethidine, 32.8% of subjects reported that pharyngeal anesthesia with Jackson spray was "painful (distress score: 4)" or "very painful (distress score: 5)", which contributed to poor tolerability by the subjects [15]. In this study, nasopharyngeal anesthesia was performed after intravenous administration of sedative/analgesic drugs. Only 2 of 50 subjects (4%) responded that nasopharyngeal anesthesia was "painful" or "very painful," and the comparison of preoperative anesthesia distress scores was significantly lower in this study than in previous reports. This was thought to be related to the fact that sedatives were administered intravenously first, and then nasal anesthesia was administered when the patient was unconscious, thereby reducing invasiveness.

Cough frequency

In the present study, the cough frequency was found to be significantly lower in the TNB group than in the TOB group. A previous study reported that the subjects were female, the examination time was long, and the procedure was EBUS-TBNA were independent factors causing strong cough in bronchoscopy [16]. Although there are no reports examining the difference in cough frequency between TNB and TOB, there were no significant differences in the frequency of gender, procedure content, or examination time between these two groups in this study, suggesting that transnasal endoscopy may have contributed to a reduction in the cough frequency. In addition, there is a previous report that droplets could be suppressed by passing the scope through a mask with holes [17]. Therefore, the combined use of transnasal bronchoscopy and surgical masks may further suppress the spread of droplets and is expected to be a measure against aerosol generation during the examination.

Limitations

This study was conducted on a small number of patients at a single institution, and the choice of transnasal or oral, and the type of scope was made at the discretion of the attending physician which may have caused a case selection bias. Also, the amount of anesthesia and frequency of coughing could have varied depending on the timing of bronchoscopies. Further analysis of the factors that suppress cough frequency will require accumulation of more cases, and a randomized controlled trial of nasal and oral administration using the same scope is necessary.

## Conclusions

The TNB under a surgical mask was comparable to the tolerability of TOB, and omission of pharyngeal anesthesia with Jackson spray was well tolerated. Regarding cough frequency, TNB was significantly lower than TOB and was further reduced by using a thin diameter scope. Nasal bronchoscopy may contribute to the prevention of the spread of novel coronavirus infection as a measure against aerosol generation.
